# Supplementary material for: scMoMaT jointly performs single cell mosaic integration and multi-modal bio-marker detection
Source: Nat Commun. 2023 Jan 24;14:384. doi: 10.1038/s41467-023-36066-2 (PMC9873790; doi:10.1038/s41467-023-36066-2)
Supplement: Supplementary file 1 — Supplementary Information [file 41467_2023_36066_MOESM1_ESM.pdf]

Supplementary Information

Supplementary Figures and tables

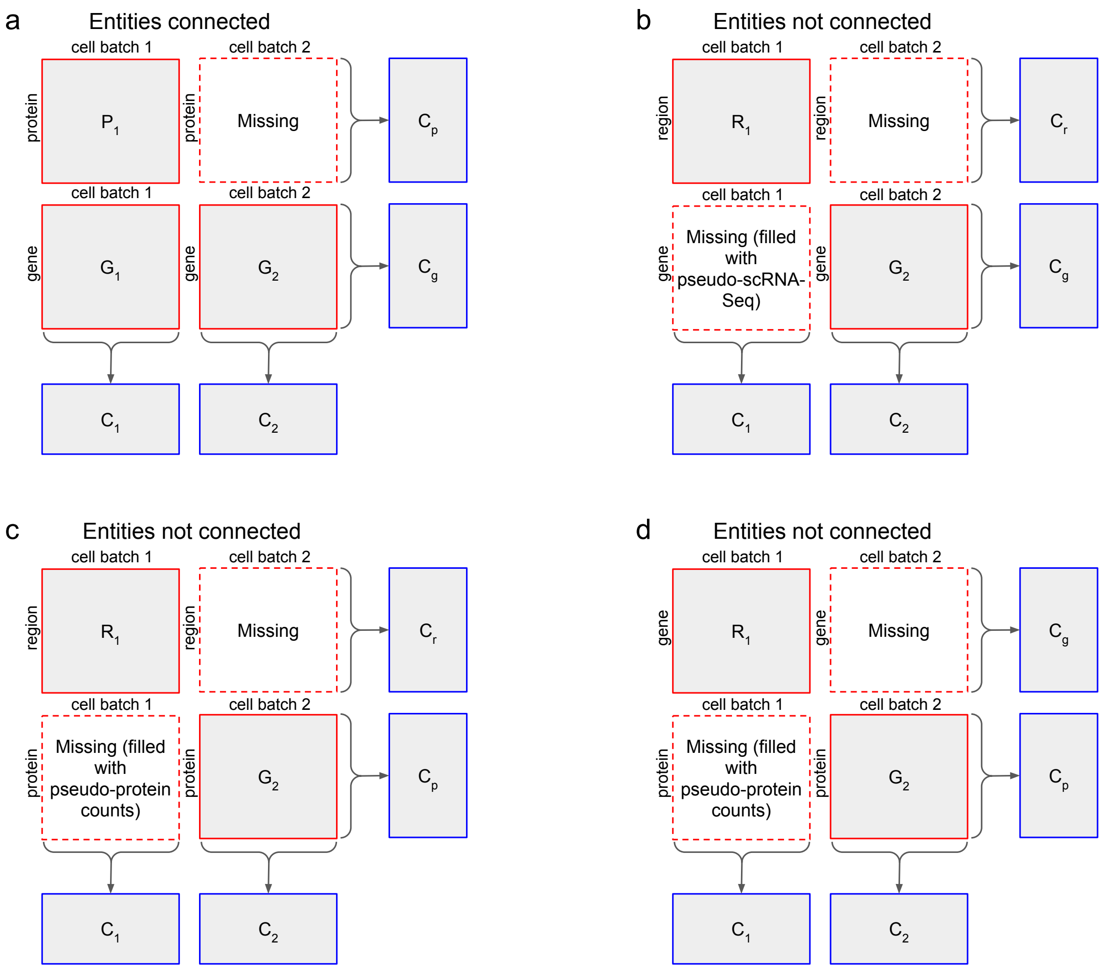

Supplementary Figure 1. Two example integration scenarios of scMoMaT. **a**. An example where data batches have common modality. **b-d**. Examples where data batches do not have common modality. scMoMaT fills in the missing modality using (**b**) pseudo-scRNA-seq matrix, and (**c-d**) pseudo-protein count matrix, and jointly factorizes the data matrices along with pseudo-count matrix.

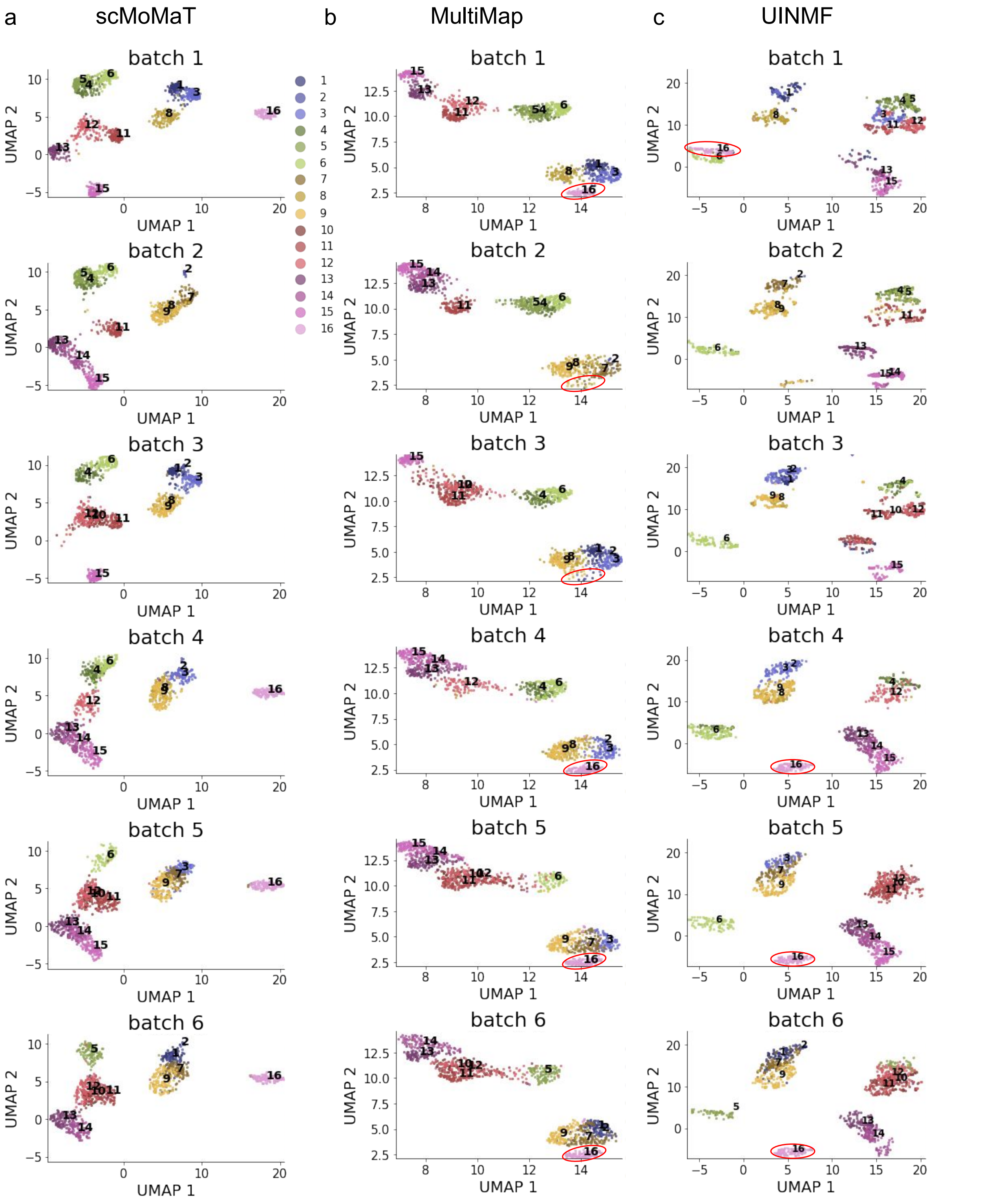

Supplementary Figure 2. Cell embedding of scMoMaT, MultiMap, and UINMF on one example simulated dataset, visualized using UMAP. For each method, cells are plot separately for each batch, and colored by ground truth cell type labels. **a**. The cell embedding of scMoMaT; **b**. The cell embedding of MultiMap; **c**. The cell embedding of UINMF. Source data are provided in the Source Data file.

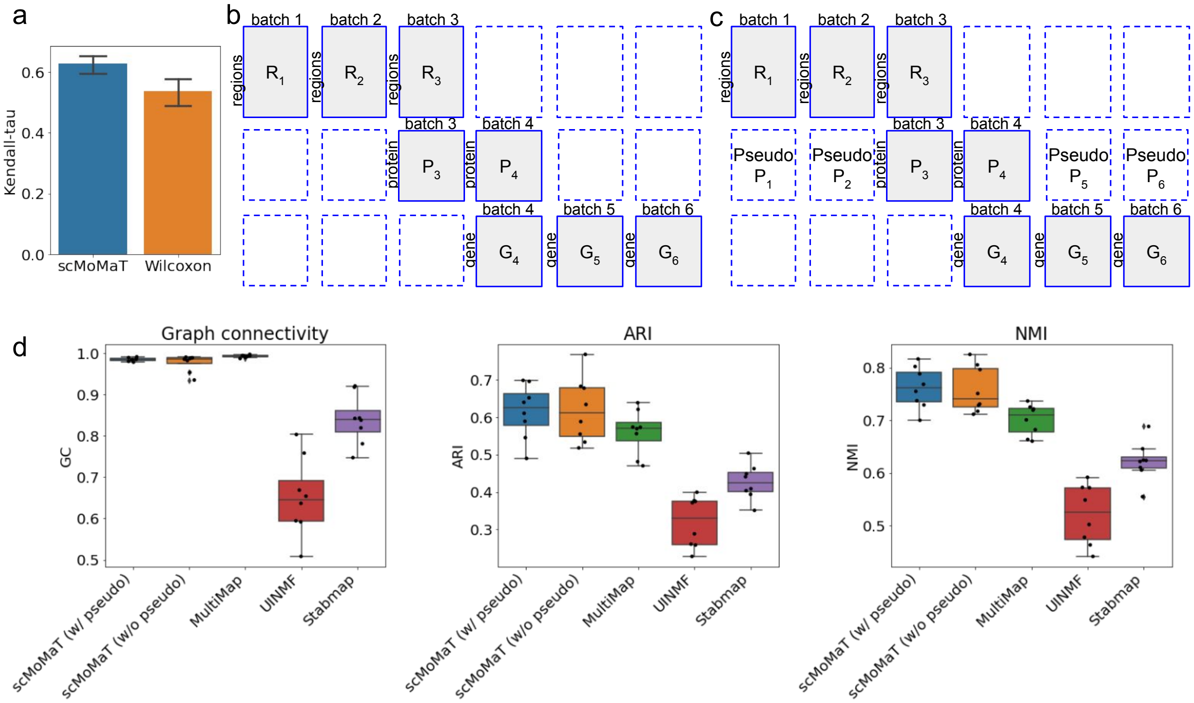

Supplementary Figure 3. Additional test results on simulated datasets. **a**. Scores of scMoMaT and the baseline method ("Wilcoxon" means first performing UINMF and then using Wilcoxon rank sum test to detect DE genes) on marker genes detection. $n = 51$ samples are included in the bar of scMoMaT, and $n = 60$ samples are included in the bar of Wilcoxon. **b-c**. The layout of data matrices under the three-modality integration scenario (**b**) without pseudo-protein counts and (**c**) with pseudo-protein counts. **d**. The GC, ARI, and NMI scores of scMoMaT with pseudo-protein counts (scMoMaT (w/ pseudo)), scMoMaT without pseudo-protein counts (scMoMaT (w/o pseudo)), MultiMap, UINMF, and StabMap. In the boxplots, the center lines show the median data value, and the box limits show the lower and upper quartiles (25% and 75%, respectively). The length of the whiskers is within 1.5x interquartile range. Outliers beyond the whiskers are plotted as points. $n = 8$ independent samples are included in each box. Source data for a and d are provided in the Source Data file.

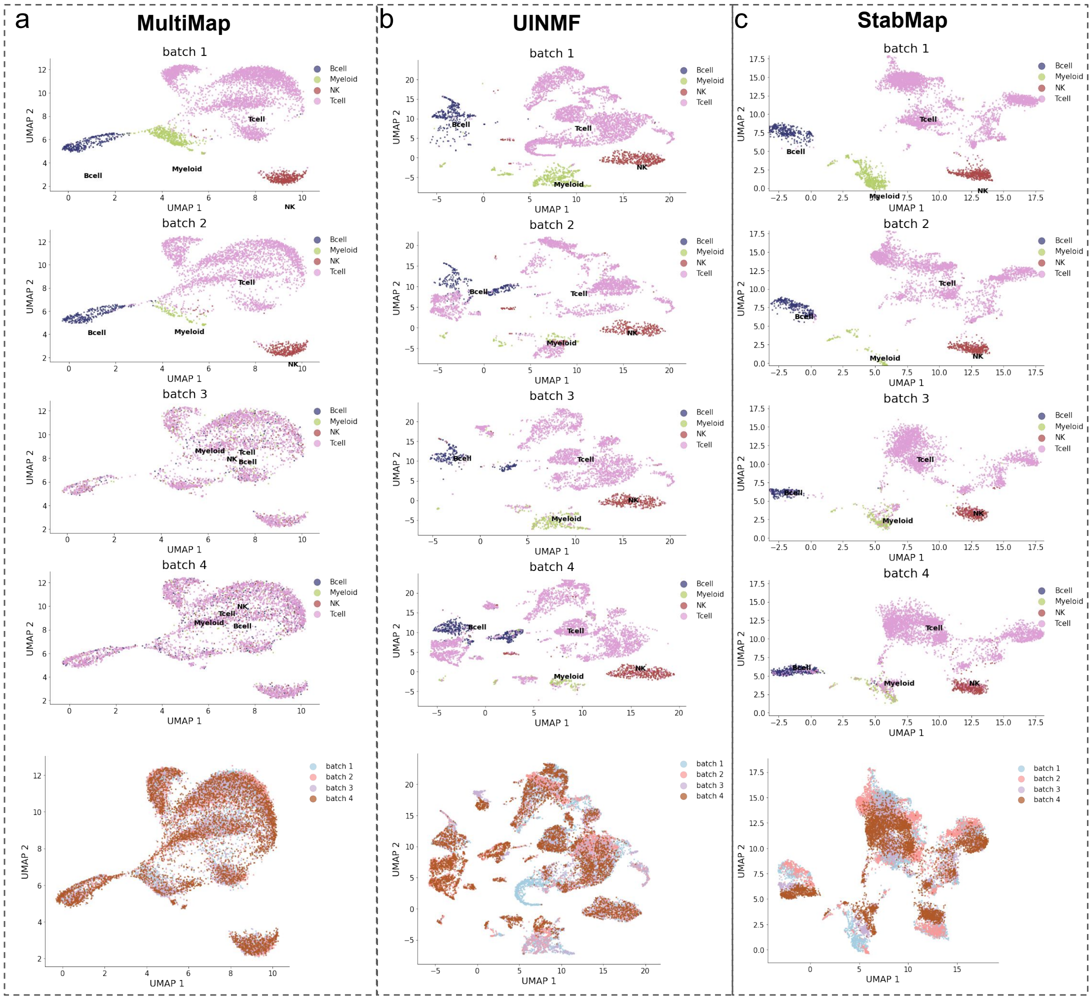

Supplementary Figure 4. Cell embedding of MultiMap, UINMF, and StabMap on the human PBMC dataset, visualized using UMAP. **a**. (Upper four plots) The cell embedding of MultiMap, where cells are plot separately for different batches, and colored by cell type labels from original data paper. (Lower plot) The cell embedding of MultiMap, where cells are colored by data batches. **b**. (Upper four plots) The cell embedding of UINMF, where cells are plot separately for different batches, and colored by cell type labels from original data paper. (Lower plot) The cell embedding of UINMF, where cells are colored by data batches. **c**. (Upper four plots) The cell embedding of StabMap, where cells are plot separately for different batches, and colored by cell type labels from original data paper. (Lower plot) The cell embedding of StabMap, where cells are colored by data batches. Source data are provided in the Source Data file.

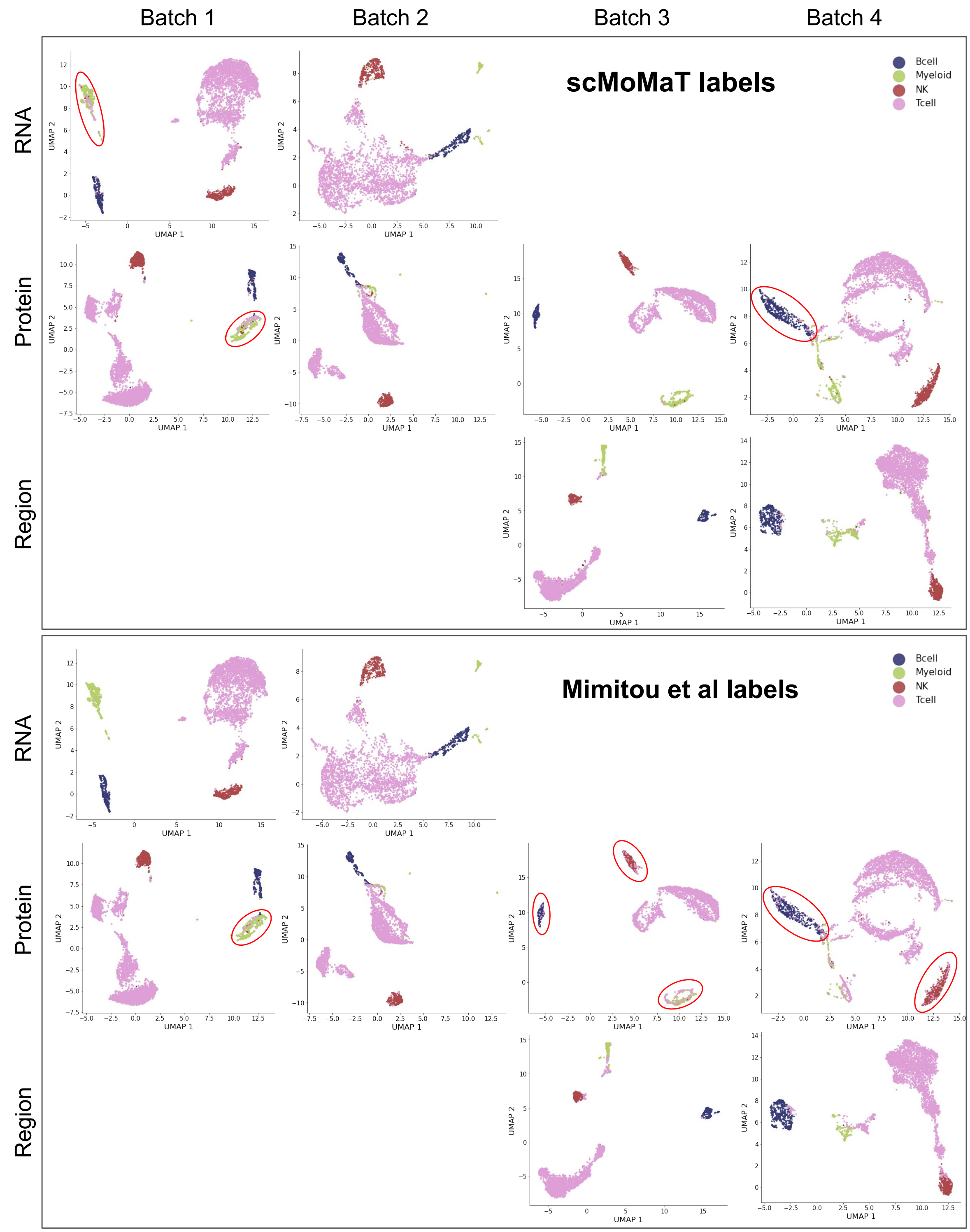

Supplementary Figure 5. UMAP visualization of each of the 8 data matrices in human PBMC dataset, where cells are colored by the cell type label from scMoMaT, and the cell type label in the original data paper (Mimitou *et al*).

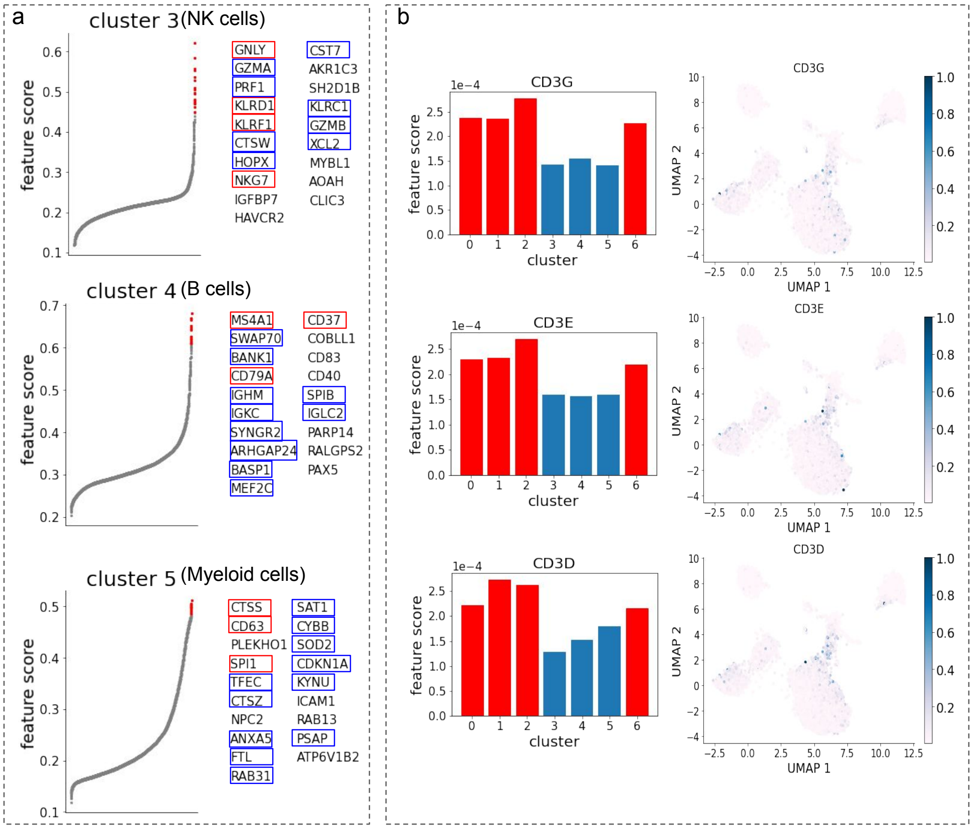

Supplementary Figure 6. Additional results on human PBMC dataset. **a**. The top-20 scoring genes in cluster 3 (NK cells), 4 (B cells), and 5 (Myeloid cells). Known marker genes are annotated in red and blue frames. **b**. (Left) The scores of T cell marker genes *CD3G*, *CD3E*, and *CD3D* of different Leiden clusters, where x-axis corresponds to Leiden clusters. Top-scoring clusters are colored red. (Right) Abundance levels of *CD3G*, *CD3E*, and *CD3D* on the cell embedding of scMoMaT. Source data are provided in the Source Data file.

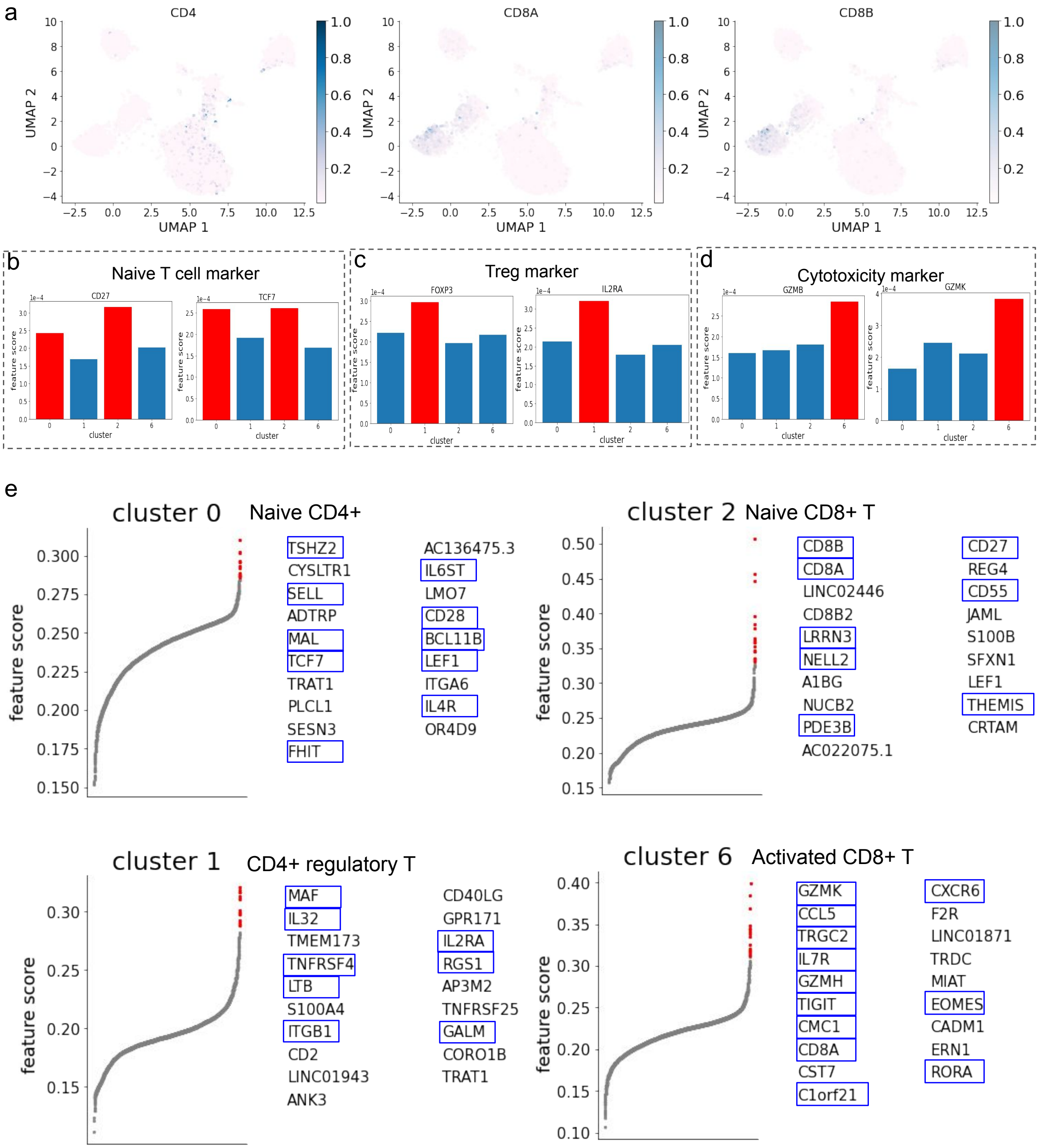

Supplementary Figure 7. Additional results on human PBMC dataset. **a**. Abundance levels of *CD4*, *CD8A*, and *CD8B* on the cell embedding of scMoMaT. **b-d**. scores of (**b**) Navie T cell marker genes, (**c**) Treg cell marker genes, and (**d**) Cytotoxicity cell marker genes in different Leiden clusters, where x-axis corresponds to Leiden clusters. **e**. The top-20 scoring genes of cluster 0 (Naive CD4+), 2 (Naive CD8+), 1 (CD4+ regulatory), and 6 (activated CD8+) T cells. Known marker genes from literature are annotated in blue frames. Source data for b-e are provided in the Source Data file.

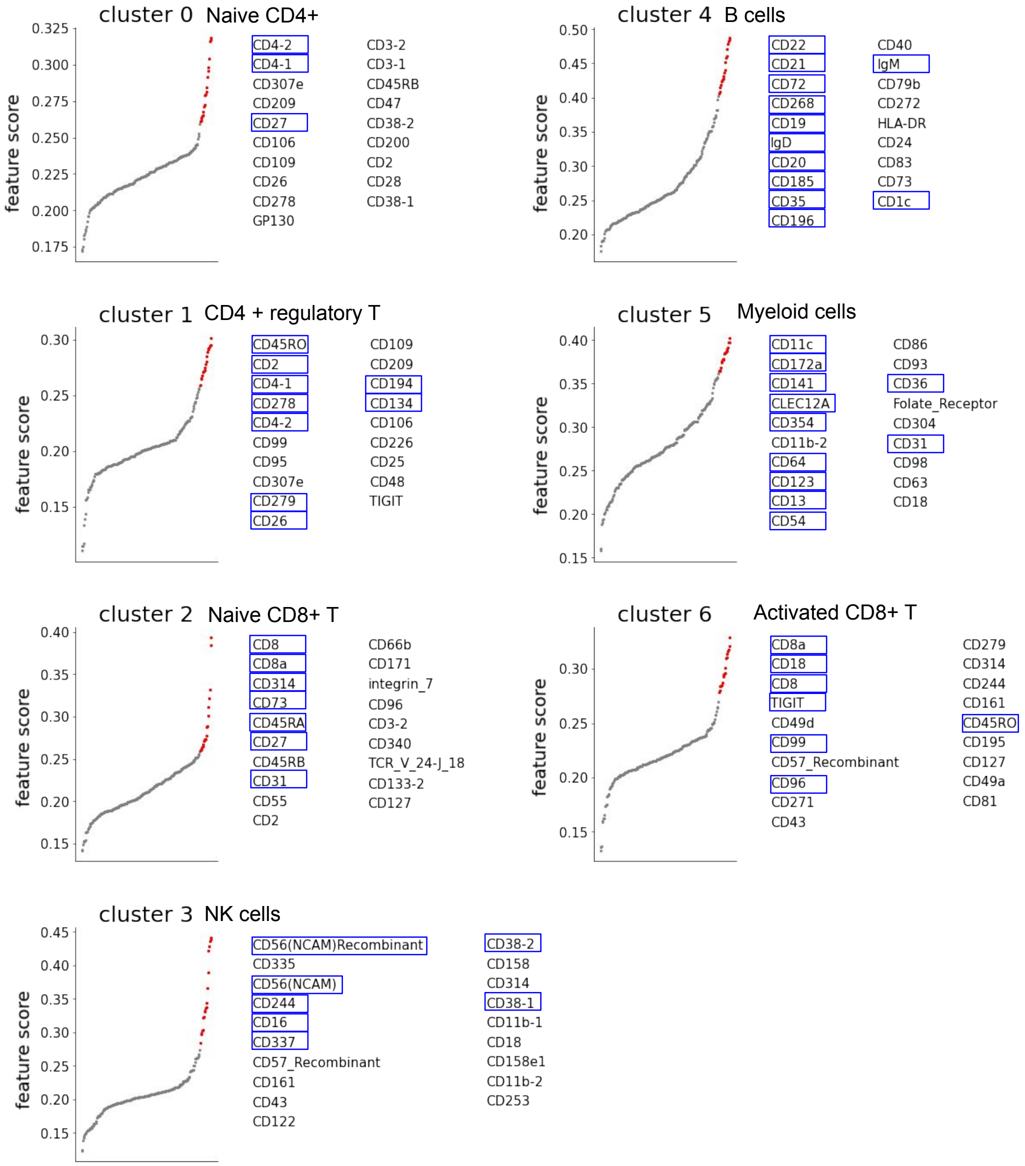

Supplementary Figure 8. Top-20 scoring proteins in all Leiden clusters of human PBMC dataset. Known marker proteins are annotated in blue frames. Source data are provided in the Source Data file.

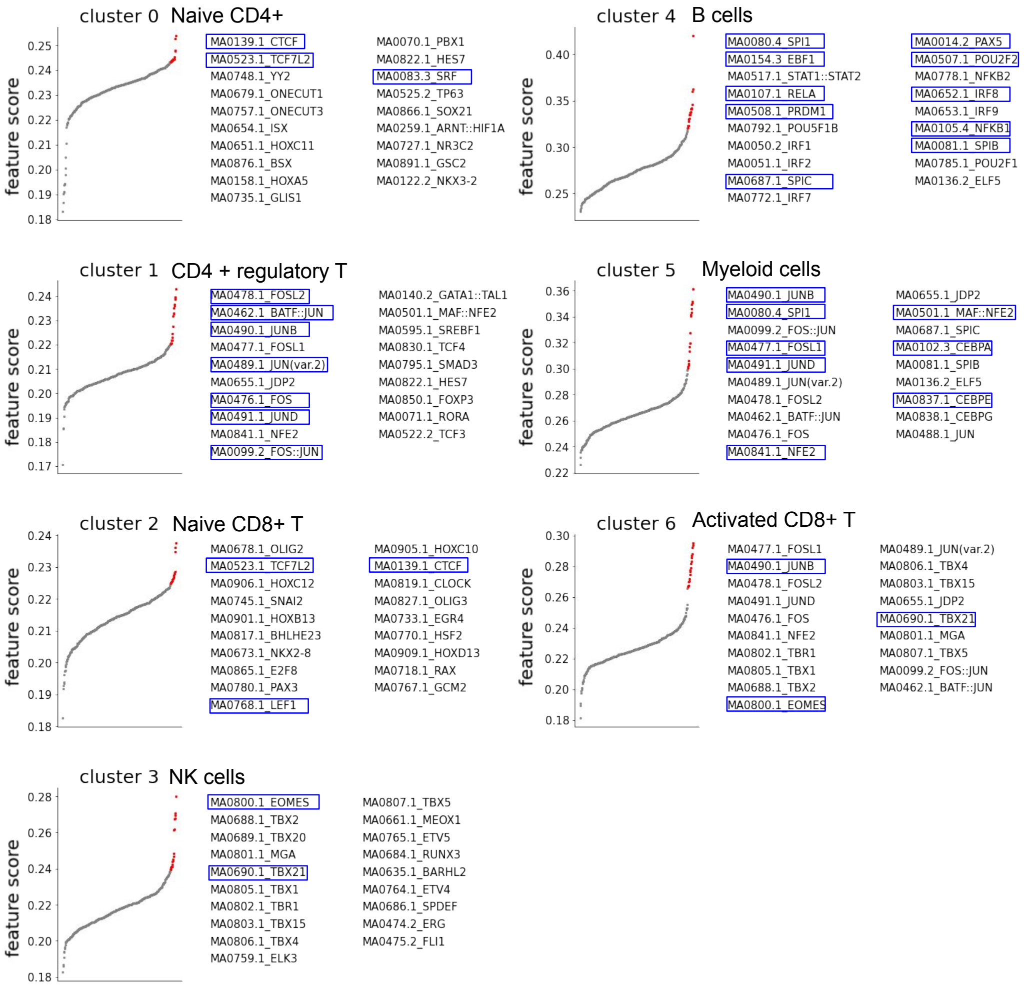

Supplementary Figure 9. Top-20 scoring motifs in all Leiden clusters of human PBMC dataset. Marker motifs corresponding to know TFs for each cell type are annotated in blue frames. Source data are provided in the Source Data file.

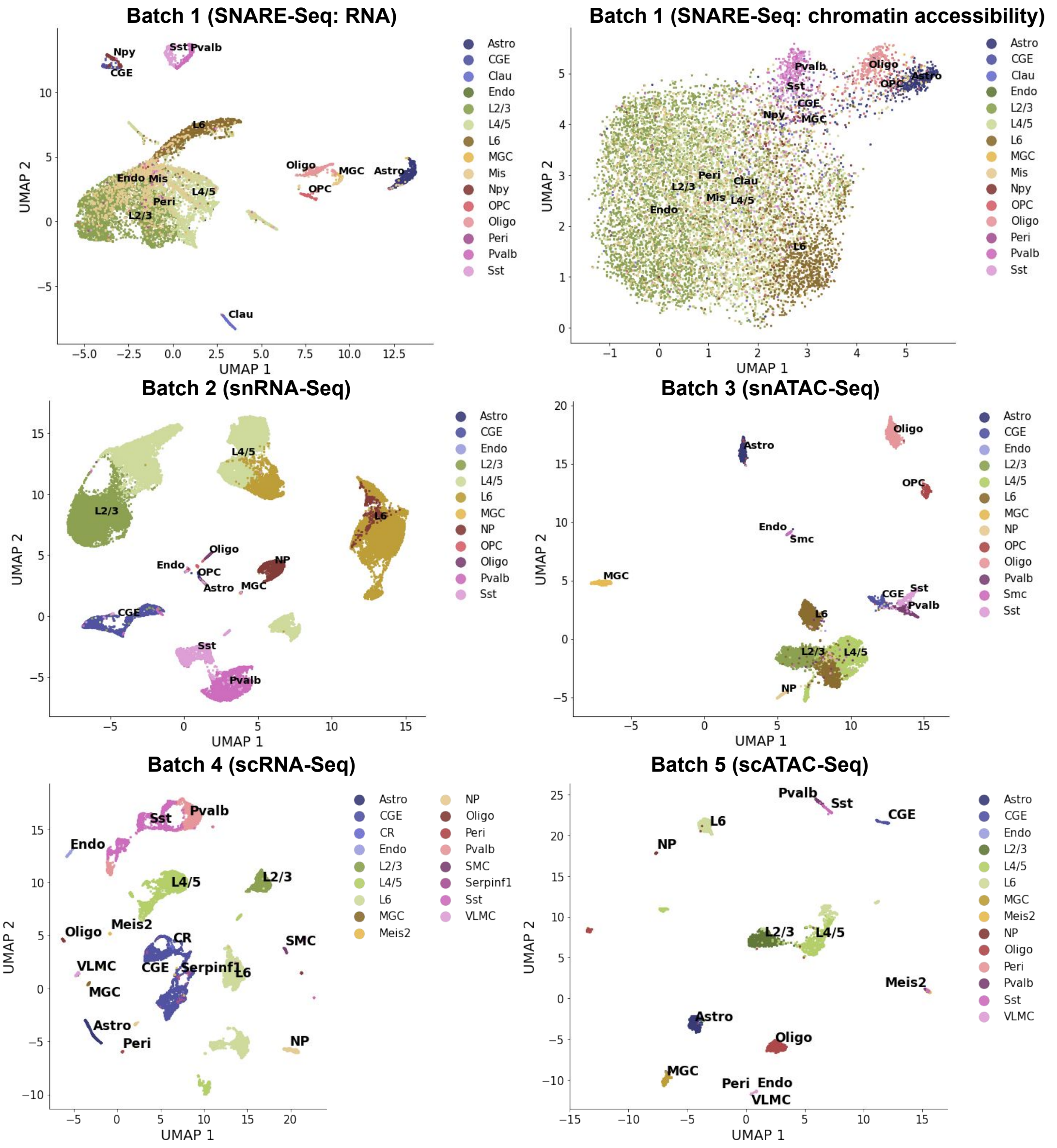

Supplementary Figure 10. UMAP visualization of 6 data matrices in mouse brain cortex dataset. Cells are colored by the cell types re-organized from the original data paper.

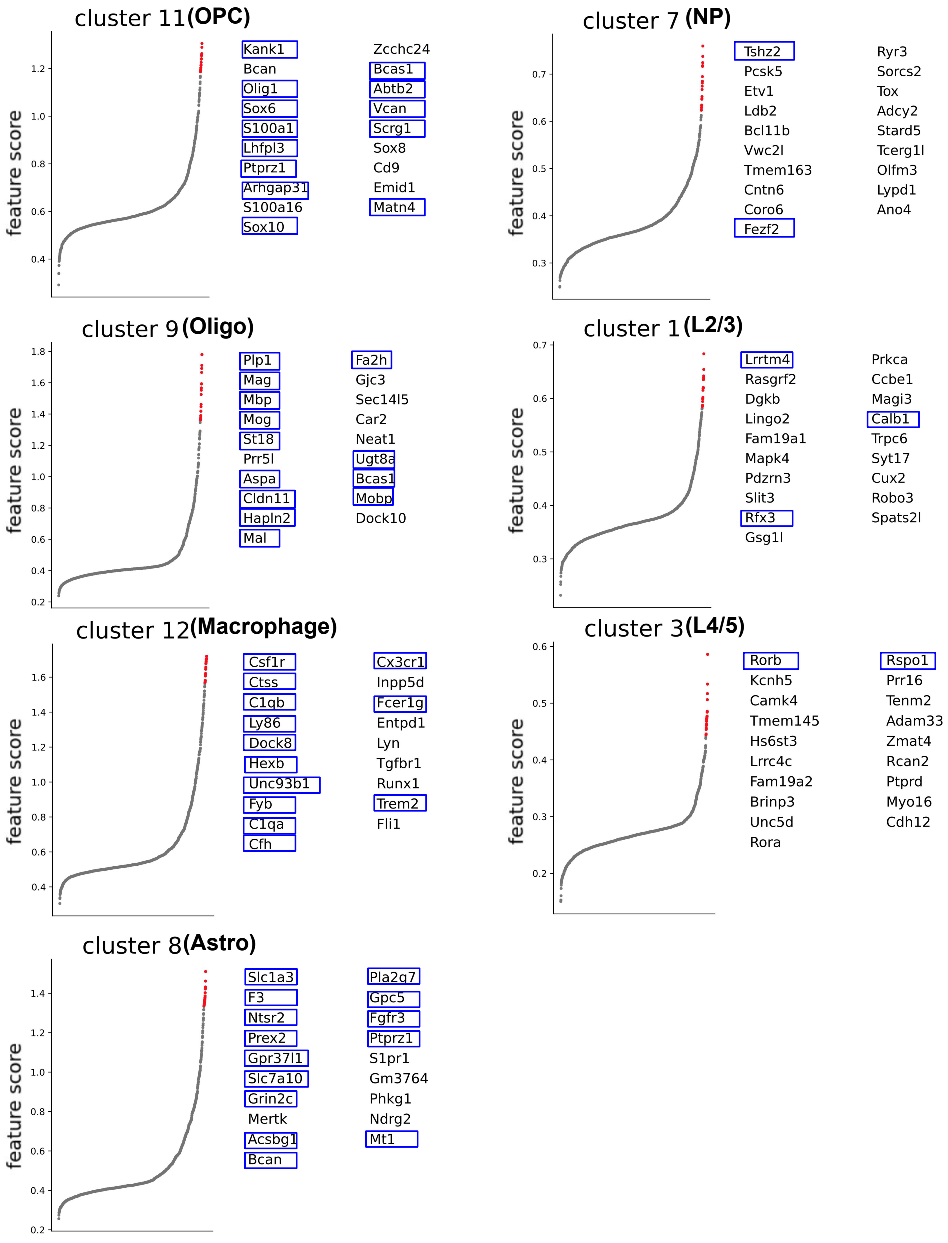

Supplementary Figure 11. Top-20 scoring genes in Leiden clusters 11 (OPC), 9 (Oligo), 12 (Macrophage), 8 (Astro), 7 (NP), 1 (L2/3), 3 (L4/5) of mouse brain cortex dataset. Known marker genes are annotated in blue frames. Source data are provided in the Source Data file.

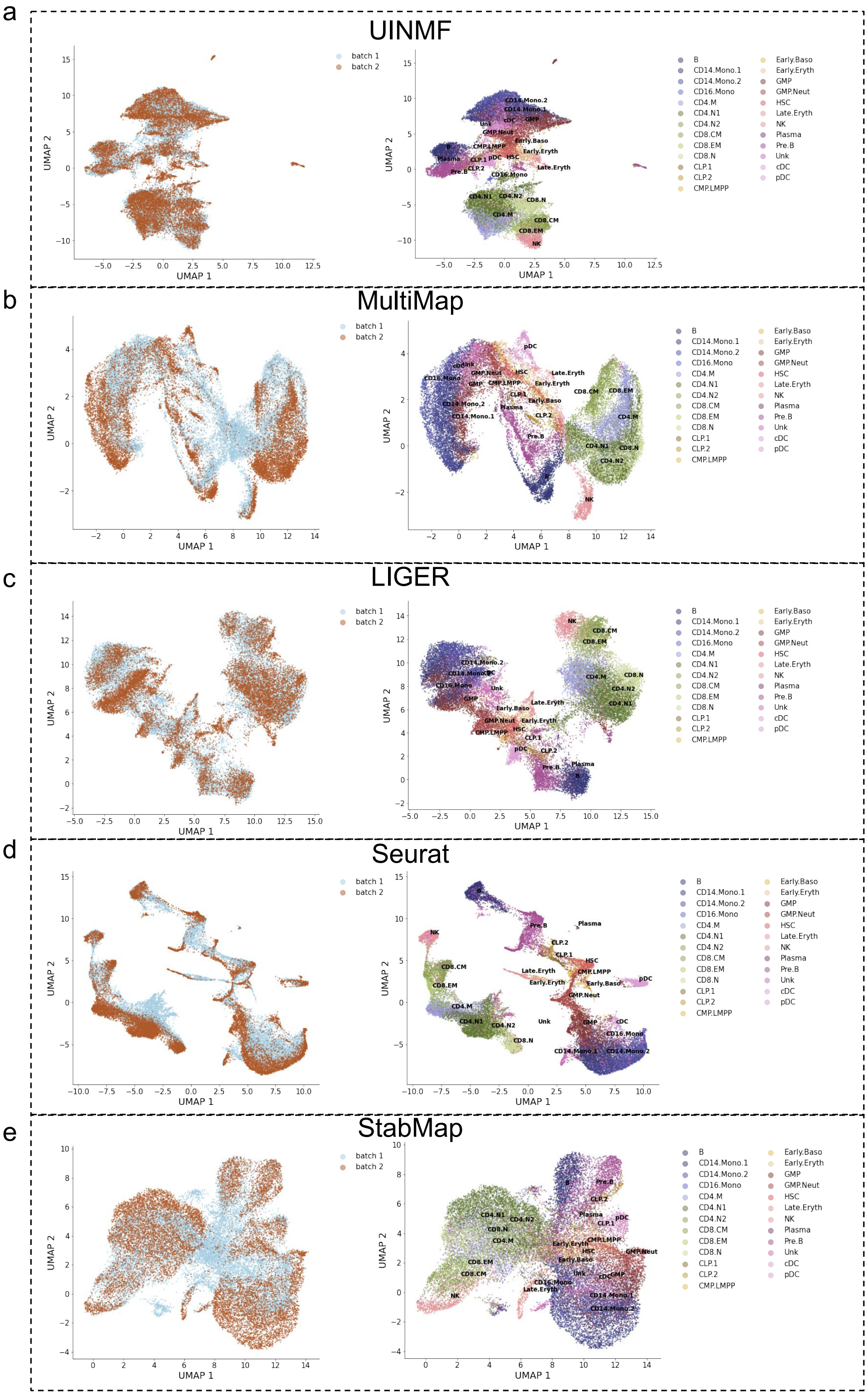

Supplementary Figure 12. Cell embedding of baseline methods for human bone marrow dataset. **a-e**. Cell embedding of (**a**) UINMF, (**b**) MultiMap, (**c**) LIGER, (**d**) Seurat, and (**e**) StabMap for human bone marrow dataset, where cells are colored by (left) data batches, and (right) cell type labels from original data paper. Source data are provided in the Source Data file.

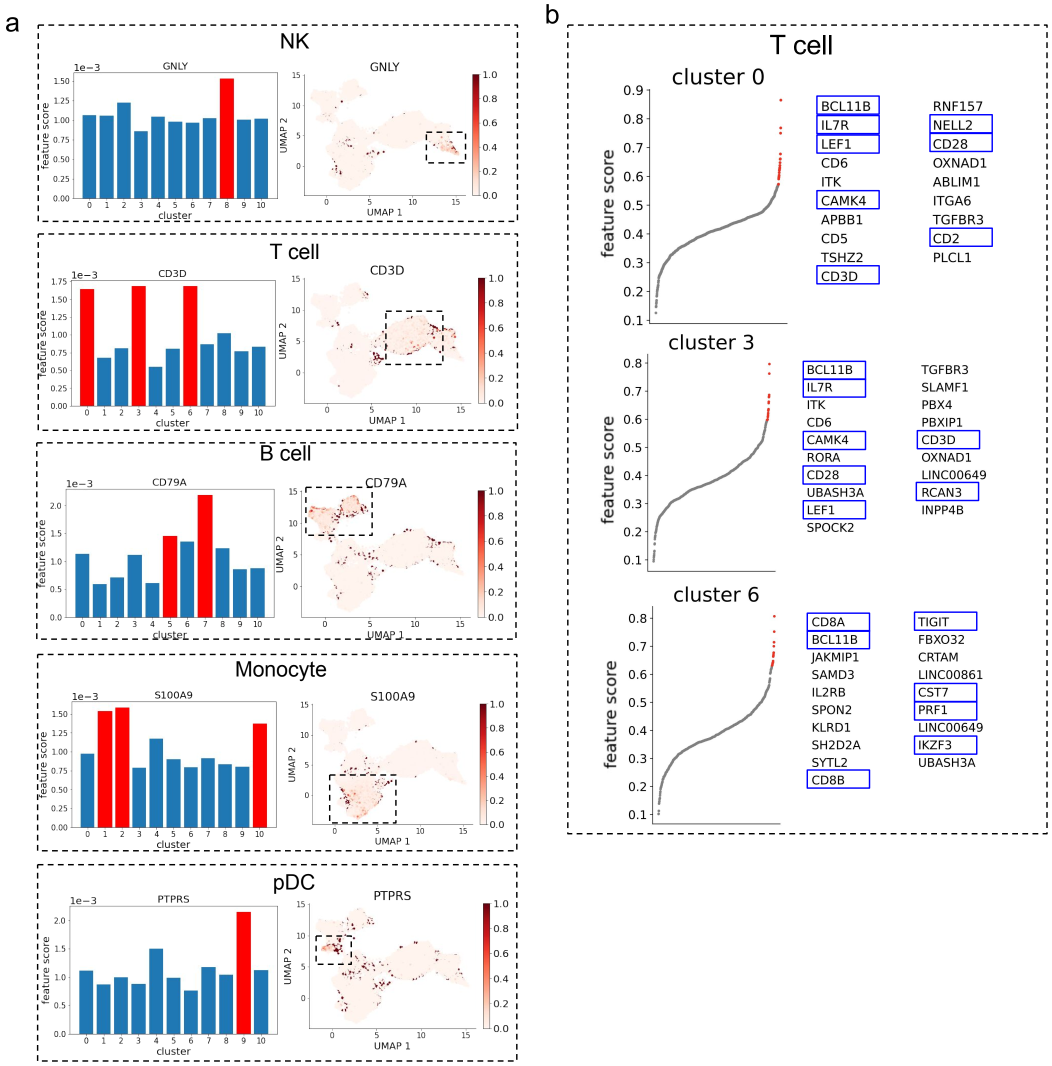

Supplementary Figure 13. Results on the gene factors learned from human bone marrow dataset. **a**. (Left) The scores of NK, T, B, Monocyte and pDC cell marker genes in different Leiden clusters, where x-axis correspond to Leiden clusters. (Right) Abundance level of these marker genes on cell embedding learned from scMoMaT. **b**. The top-20 scoring genes of the Leiden clusters that correspond to T cell (cluster 0, 3, and 6). Known marker genes are annotated in blue frames. Source data are provided in the Source Data file.

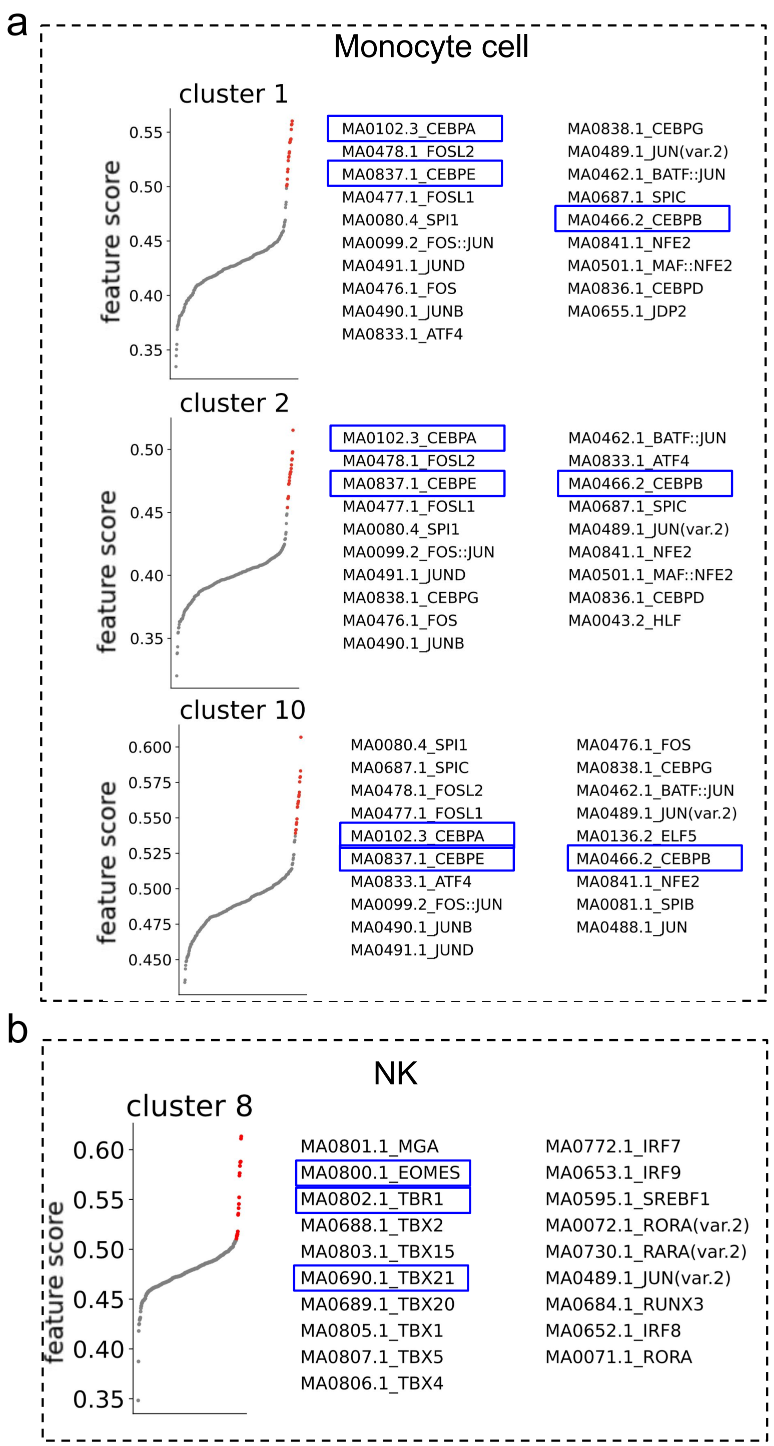

Supplementary Figure 14. The motif factors learned from human bone marrow dataset. **a**. The top-20 scoring motifs of the Leiden clusters that correspond to Monocyte cells (cluster 1, 2, and 10), where known marker motifs are annotated in blue frames. **b**. The top-20 scoring motifs of the Leiden cluster that correspond to NK cells (cluster 8), where known marker motifs are annotated in blue frames. Source data are provided in the Source Data file.

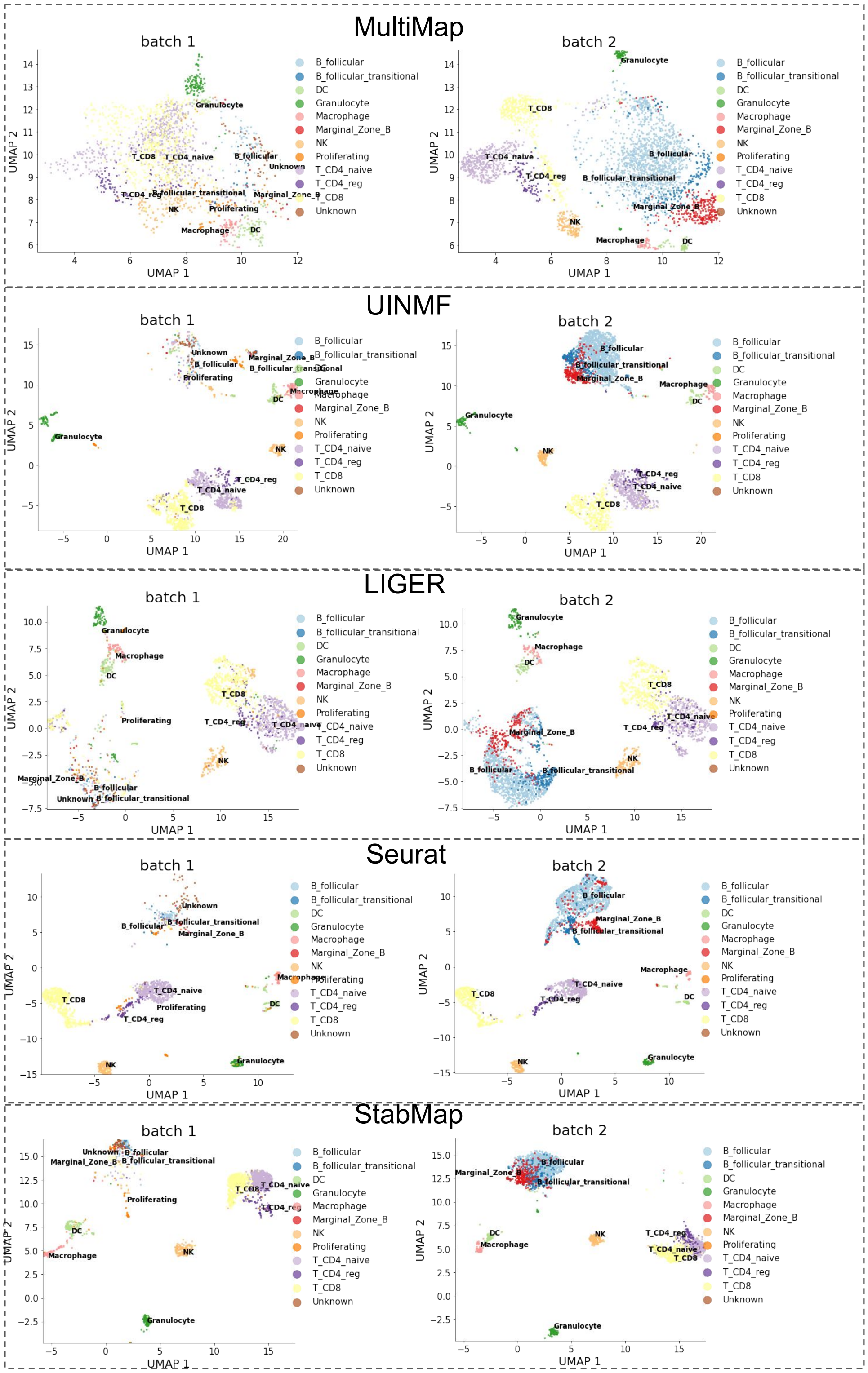

Supplementary Figure 15. Cell embedding of MultiMap, UINMF, LIGER, Seurat, and StabMap for mouse spleen dataset (after sub-sampling). Cells are colored by cell type annotation in the original data paper. Source data are provided in the Source Data file.

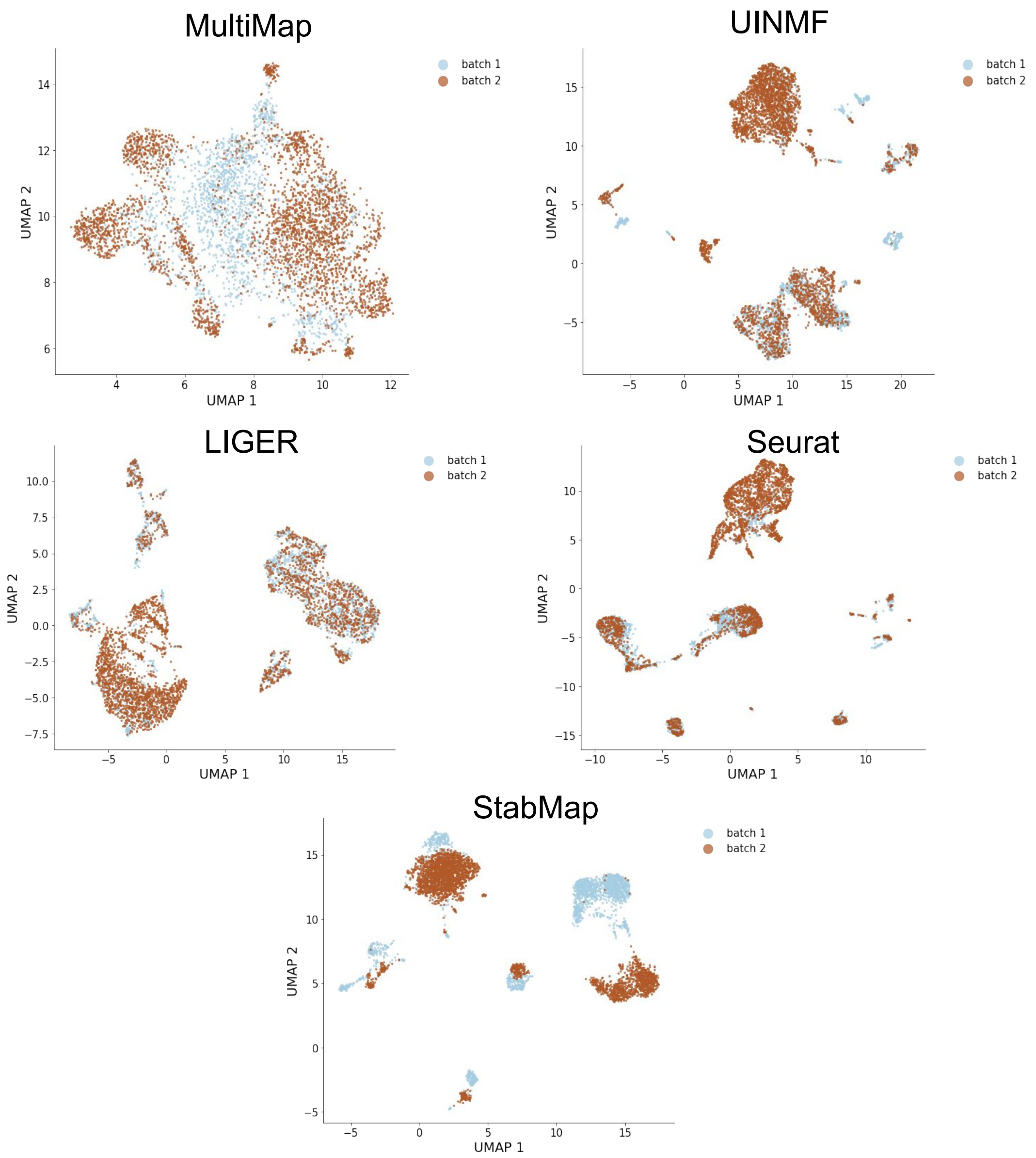

Supplementary Figure 16. Cell embedding of MultiMap, UINMF, LIGER, Seurat, and StabMap for mouse spleen dataset (after sub-sampling). Cells are colored by batches. Source data are provided in the Source Data file.

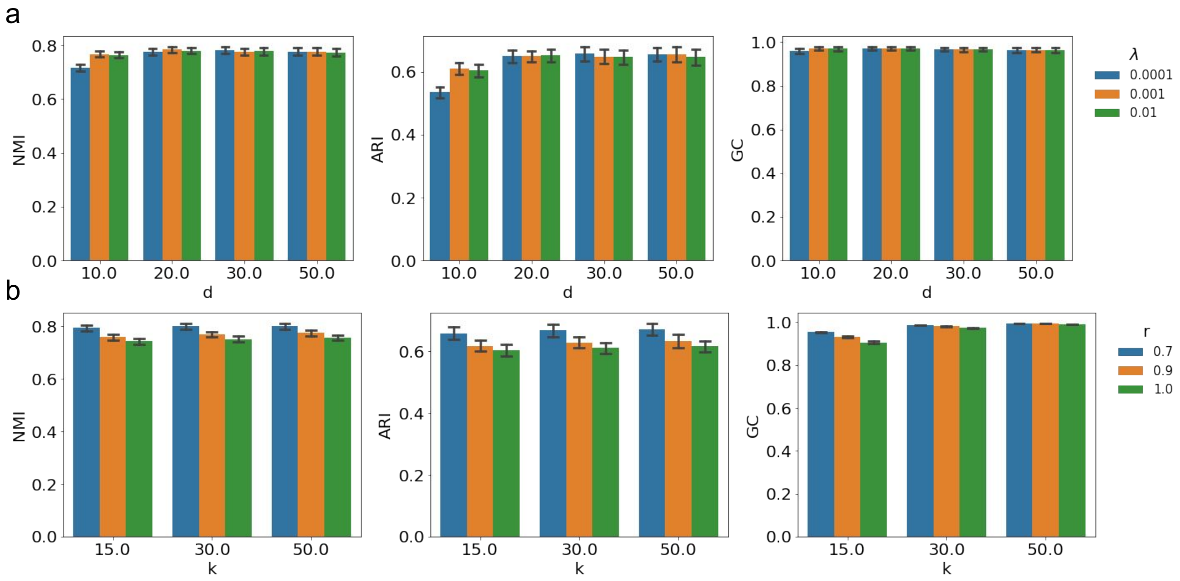

Supplementary Figure 17. Test results of hyper-parameter settings on simulated datasets. **a**. The NMI, ARI, and GC scores of scMoMaT under different latent dimensions d and regularization weights λ. $n = 45$ independent samples are included in each bar. **b**. The NMI, ARI, and GC scores of scMoMaT under different numbers of neighbors k and radius parameters r. $n = 60$ independent samples are included in each bar. In the barplots above, the error bar represent 95% confidence interval, and the center of the error bar shows the mean. Source data are provided in the Source Data file.

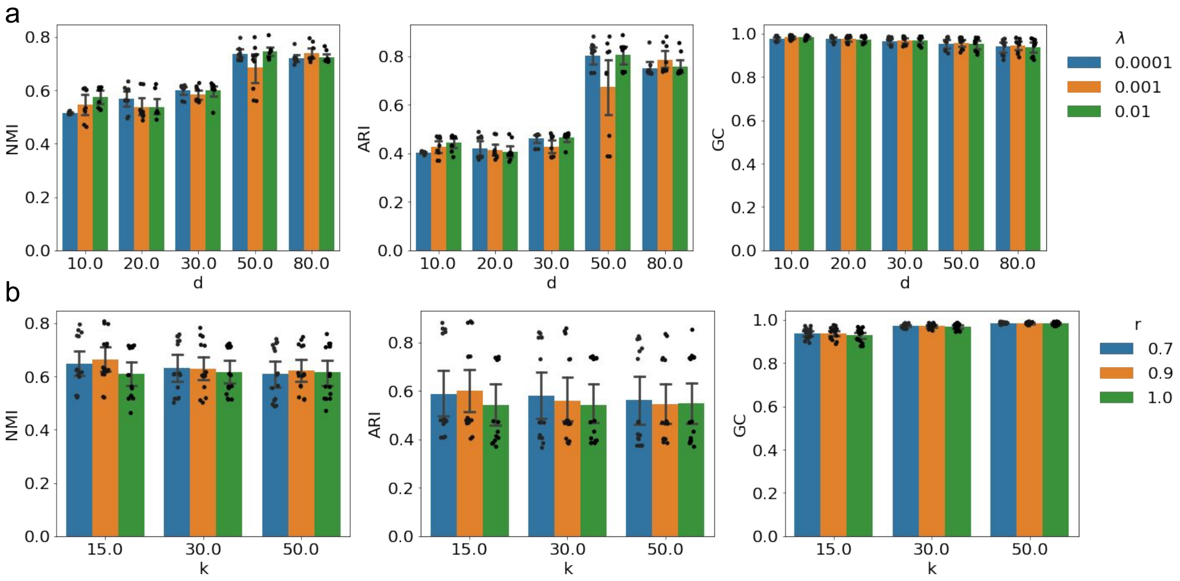

Supplementary Figure 18. Results of different hyper-parameter settings on the human PBMC dataset. **a**. The NMI, ARI, and GC scores of scMoMaT under different latent dimensions d and regularization weights λ. $n = 9$ independent samples are included in each bar. **b**. The NMI, ARI, and GC scores of scMoMaT under different numbers of neighbors k and radius parameters r. $n = 12$ independent samples are included in each bar. In the barplots above, the error bar represent 95% confidence interval, and the center of the error bar shows the mean. Source data are provided in the Source Data file.

	#genes	#regions	#protenis	#cells (batch1)	#cells (batch2)	#cells (batch3)	#cells (batch4)	#cells (batch5)	#cells (batch6)
**dataset 1**	1000	3000	200	1186	1117	1103	1160	1254	1111
**dataset 2**	1000	3000	200	1188	1159	1138	1044	1098	1153
**dataset 3**	1000	3000	200	1155	1115	1102	1108	1135	1146
**dataset 4**	1000	3000	200	1150	1137	1156	1157	1203	1080
**dataset 5**	1000	3000	200	1228	1155	1108	1127	1135	1081
**dataset 6**	1000	3000	200	1187	1199	1160	1133	1205	1104
**dataset 7**	1000	3000	200	1211	1196	1157	1125	1103	1087
**dataset 8**	1000	3000	200	1262	1277	1114	1168	1151	1059

Supplementary Table 1. The numbers of cells and features for each batch in each simulated dataset.

**Cell type**	**Marker genes**
L2/3	**Calb1,** Slc17a7, Slc30a3, Rfx3, Lamp5, Otof, Lrrtm4
L4/5	**Rorb,** Slc17a7, Slc30a3, Rspo1
L6 CT/b	**Sulf1**, **Foxp2,** Slc17a7, Fezf2
L6 IT	**Sulf1,** Slc17a7, Fezf2, Slc30a3
NP	**Tshz2**, Slc17a7, Fezf2, Sla2,
Astro	**Aldoc**, **Slc1a3**, Slc1a2, Sparcl1, Cst3, Apoe, Id3, Fabp7, Glul, Clu, Mfge8, Slc4a4, Mt1, Pla2g7, Gja1
Macrophage	**Csf1r**, **C1qb**, C1qa, Hexb, Fcer1g, Ctss, Lgmn, Cx3cr1, Trem2
OPC	**Lhfpl3**, **Matn4**, Olig1, Scrg1, S100a1, Pllp, Sox10, Cspg5, Ostf1
Oligo	**Plp1**, **Mbp**, Bcas1, Cnp, Mag, Cldn11, Enpp6, Cd9, Tubb4a, Lims2, Ugt8a, Mobp
GABAergic	**Pvalb**, **Sst**, **Npy**, **Vip,** Lamp5, Sncg

Supplementary Table 2. Marker gene list of different cell types in mouse brain cortex dataset, collected from literature.

Supplementary Note 1: pseudo-code of scMoMaT

Algorithm 1 scMoMaT

function SCMOMAT($\{\mathbf{G}_i\}, \{\mathbf{R}_j\}, \{\mathbf{P}_k\}$)
    Initialize $\mathbf{C}_x$, Σ, $\{\Sigma_{xx}\}$ with uniform distribution between 0 and 1, $\{\mathbf{C}_i\}$, $\{\mathbf{C}_j\}$ are then transformed by softmax function.
    for t **in** $1, 2, \cdots, T$ **do**
        //Construct mini-batch by sampling cells and features
        $\mathbf{G}_i^s, \mathbf{R}_j^s, \mathbf{P}_k^s, \mathbf{C}_x^s, \mathbf{b}_{xx}^s$ = sample_minibatch($\mathbf{G}_i, \mathbf{R}_j, \mathbf{P}_k, \mathbf{C}_x, \mathbf{b}_{xx}$)
        // loop through parameter matrices, and update them one by one using stochastic gradient descent
        for Y **in** $\{\mathbf{C}_x^s\}, \Sigma, \{\Sigma_{xx}\}$ **do**
            //L is the loss function of scMoMaT, $\nabla_{\mathbf{Y}} L$ is the gradient of L with regard to Y
            $\mathbf{Y} = \mathbf{Y} - \nabla_{\mathbf{Y}} L(\mathbf{G}_i^s, \mathbf{R}_j^s, \mathbf{P}_k^s)$
            if $Y = \Sigma$ **then**
                // make sure all elements in Σ are non-negative
                $Y = Y \odot (Y > 0)$
        for Y **in** $\{\mathbf{b}_{xx}^s\}$ **do**
            // following the closed-form solution of $\mathbf{b}_{xx}$
            $Y = \arg\min_Y L(\mathbf{G}_i^s, \mathbf{R}_j^s, \mathbf{P}_k^s)$
        for Y **in** $\{\alpha_{xx}\}$ **do**
            // following the closed-form solution of α_{xx}
            $Y = \arg\min_Y L(\mathbf{G}_i^s, \mathbf{R}_j^s, \mathbf{P}_k^s)$

Supplementary Note 2: simulating protein count from single cell gene expression counts

In our procedure to simulate multi-modality single cell data, we first generate the two modalities: gene expression and chromatin accessibility, and then generate the protein counts from the gene expression counts as follows:

(1) We first select a reference protein count dataset and fit its counts into a protein count distribution. We then can sample new protein counts from this distribution. We use the protein count in the human PBMC dataset[1] (the first real dataset in the manuscript) as the reference dataset.

(2) For each batch in the simulated datasets, we generated its protein count matrix from the corresponding gene expression matrix. As current protein abundance data matrices profile only a subset of proteins, we select the top 200 highly variable genes from the gene expression matrix, and generate counts for the corresponding 200 proteins, where each protein is associated with one gene.

(3) We assume the protein counts are positively correlated with the corresponding gene counts, and are sampled from the reference protein count distribution. We draw $200 \times n$ samples from the distribution, where n is the number of cells in the given batch. We fill these $200 \times n$ samples into the protein count matrix according to the rank of their corresponding gene expression level. To account for the technical noise within the protein counts, we randomly select 10% of the protein samples, and randomly permute their rank-based value assignment when filling them into the protein count matrix.

References

1. Mimitou, E. P. *et al.* Scalable, multimodal profiling of chromatin accessibility, gene expression and protein levels in single cells. *Nat. Biotechnol.* (2021).
