# Supplementary material for: scMoMaT jointly performs single cell mosaic integration and multi-modal bio-marker detection
Source: Nat Commun. 2023 Jan 24;14:384. doi: 10.1038/s41467-023-36066-2 (PMC9873790; doi:10.1038/s41467-023-36066-2)
Supplement: Supplementary file 3 — Description of additional Supplementary File [file 41467_2023_36066_MOESM3_ESM.pdf]

Descriptions of additional supplementary files

Supplementary Data 1: Motif markers and their corresponding sources in the human PBMC dataset.
